# Prevention of infective endocarditis in at-risk patients: how should dentists proceed in 2024?

**DOI:** 10.1038/s41415-024-7355-2

**Published:** 2024-05-10

**Authors:** Martin Thornhill, Bernard Prendergast, Mark Dayer, Ash Frisby, Peter Lockhart, Larry M. Baddour

**Affiliations:** 4141556428001 Professor of Translational Research in Dentistry, Unit of Oral and Maxillofacial Medicine, Surgery and Pathology, School of Clinical Dentistry, University of Sheffield, Sheffield, UK; 4141556428002 Professor of Cardiology, Guy´s and St Thomas´ Hospital, London and Chair of Cardiology, Cleveland Clinic, London, UK; 4141556428003 Professor and Consultant Cardiologist, Cardiovascular Research Institute, Mater Private Network, Dublin, Ireland; 4141556428004 Patient Advocate, London, UK; 4141556428005 Research Professor, Department of Oral Medicine/Oral and Maxillofacial Surgery, Atrium Health´s Carolinas Medical Centre, Charlotte, North Carolina, USA; Adjunct Professor, Department of Otolaryngology, Wake Forest University School of Medicine, North Carolina, USA; 4141556428006 Professor Emeritus, Division of Public Health, Infectious Diseases and Occupational Health, Departments of Medicine and Cardiovascular Medicine, Mayo Clinic College of Medicine and Science, Rochester, Minnesota, USA

## Abstract

National Institute for Health and Care Excellence (NICE) guidelines are ambiguous over the need for patients at increased risk of infective endocarditis (IE) to receive antibiotic prophylaxis (AP) prior to invasive dental procedures (IDPs), and this has caused confusion for patients and dentists alike. Moreover, the current law on consent requires clinicians to ensure that patients are made aware of any material risk they might be exposed to by any proposed dental treatment and what can be done to ameliorate this risk, so that the patient can decide for themselves how they wish to proceed. The aim of this article is to provide dentists with the latest information on the IE-risk posed by IDPs to different patient populations (the general population and those defined as being at moderate or high risk of IE), and data on the effectiveness of AP in reducing the IE risk in these populations. This provides the information dentists need to facilitate the informed consent discussions they are legally required to have with patients at increased risk of IE about the risks posed by IDPs and how this can be minimised. The article also provides practical information and advice for dentists on how to manage patients at increased IE risk who present for dental treatment.

## Key points

- This paper provides dentists with the information they need for informed consent discussions with patients about the risk of developing infective endocarditis following invasive dental procedures.
- Gives dentists the information they need for informed consent discussions with patients about the effectiveness of antibiotic prophylaxis in reducing the risk of infective endocarditis following invasive dental procedures.
- Presents dentists with practical information and advice regarding the management of patients at increased risk of infective endocarditis who present to them needing dental treatment.

## Introduction

When the National Institute for Health and Care Excellence (NICE) added the word ‘routinely' to their guidance - ‘antibiotic prophylaxis against infective endocarditis is not routinely recommended for people undergoing dental procedures' - in 2016,^1^ it became clear that there were ‘non-routine' situations when antibiotic prophylaxis (AP) would be recommended. Indeed, in a letter confirming the addition of the word ‘routinely,' Sir Andrew Dillon (CEO of NICE) asserted, ‘this amendment should now make clear that in individual cases, AP may be appropriate'.^2^ The problem was that the guidelines did not clarify in which patients, and for which dental procedures, AP might be appropriate (or what AP regimen should be used).

In 2015, the law on informed consent changed following a Supreme Court judgment in the case of Montgomery vs Lanarkshire Health Board.^3^^,^^4^^,^^5^^,^^6^ As a consequence, doctors and dentists are now required to ensure that patients are aware of any ‘material risks' involved in any proposed treatment and of reasonable alternatives. Having provided this information, it is then for the patient (not the clinician) to decide which treatment they want. Importantly, this decision legally enforces principles that were already recommended by the General Dental Council, General Medical Council (Consent: patients and doctors making decisions together, 2008) and most medical/dental indemnity insurers.^7^^,^^8^

The problem was the scant evidence quantifying the risk of developing infective endocarditis (IE) after invasive dental procedures (IDPs) that dentists could use to inform patients, and the complete absence of evidence to inform patients of the potential risks and benefits of AP. However, new evidence has emerged in recent years (see accompanying article in this issue)^9^ and provides risk-related data that can be used to inform patients.

## What does the new evidence mean for your patients?

### Which dental procedures pose a risk?

IE develops when pathogenic bacteria enter the circulation of individuals with predisposing cardiac conditions and colonise the endocardial surface of the heart (particularly the heart valves) leading to the development of heart valve vegetations, valve destruction (perforations and scarring) and perivalvular abscesses. Several oral bacterial species have the potential to cause IE, including oral viridans group streptococci (OVGS), HACEK organisms (Haemophilus spp., Aggregatibacter actinomycetemcomitans, Cardiobacterium hominis, Eikenella corrodens and Kingella kingae) and some enterococci (as well as some non-bacterial organisms and fungi). Any dental procedure that enables oral flora to enter the circulation and cause a bacteraemia should therefore be considered an IDP. These are largely procedures closely associated with the gingival or periapical region of the teeth that can result in bleeding. Certain procedures, such as extractions, oral surgery, scaling and endodontic interventions, frequently cause bacteraemia and should always be considered as IDPs.^10^ Some restorative and orthodontic procedures (eg crown or subgingival cavity preparations, placement of matrix bands or tooth separators etc) may also result in bacteraemia and should be considered IDPs if any gingival manipulation or bleeding is anticipated, while other restorative or orthodontic procedures (eg preparation and placement of restorations that do not involve the gingiva) do not.

At one time, guideline committees produced exhaustive lists of invasive and non-invasive dental procedures but soon realised that such lists were over-prescriptive and unhelpful. Only dentists themselves know whether a planned procedure has potential to cause bacteraemia. Hence, major guideline committees (including the European Society of Cardiology [ESC] and American Heart Association [AHA]) have adopted almost identical definitions for IDPs (Box 1) that are simple for dentists to understand and implement.

Box 1 Dental procedures (based on 2021 AHA and 2023 ESC guidelines)^19^^,^^24^ IDPs:

All dental procedures that involve manipulation of the gingival tissue or the periapical region of the teeth or perforation of the oral mucosa. This includes all extractions, oral surgical procedures (including periodontal surgery, implant surgery and oral biopsies), scaling and root canal procedures. It also includes restorative and orthodontic procedures that involve manipulation (or bleeding) of the gingival tissues or periapical region of the teeth, or perforation of the oral mucosa.

Not considered IDPs:

- Anaesthetic injections through non-infected tissue
- Dental x-rays
- Placement of removable prosthodontic or orthodontic appliances
- Adjustment of orthodontic appliances
- Placement of orthodontic brackets
- Shedding of primary teeth
- Bleeding from trauma to the lips or oral mucosa.

### Which patients are at increased risk of IE?

There is a clear consensus as to which cardiac conditions predispose an individual to IE and its adverse outcomes (see Table 1 in accompanying article).^9^ Most of the population without predisposing cardiac conditions are considered at low/unknown IE risk and bacteraemia caused by an IDP is unlikely to result in IE. However, certain cardiac conditions place an individual at high risk of IE (including a previous history of IE, the presence of a prosthetic or repaired heart valve, and certain congenital heart conditions), whilst other groups are at moderate (or intermediate) risk (see Table 1 in accompanying article).^9^ Before 2007, AP was recommended for all patients at increased risk (moderate and high), but since then, most guideline committees around the world have recommended restriction of AP to those at high risk. This represented a 90% reduction in the size of the population for whom AP is recommended and a substantial reduction in unnecessary antibiotic use. NICE, however, recommended complete cessation of AP in 2008 (and again in 2015)^1^^,^^11^ and the rationale for these changes (and accompanying controversy) is fully described in an accompanying article in this journal.^9^

Dentists are seeing increasing numbers of patients present for treatment that have coronary artery stents that were inserted to treat coronary artery disease, including angina or myocardial infarction. However, infection of these, and other vascular stents, is extremely rare and, when it occurs, is nearly always the result of staphylococcal infection originating from the skin (rather than bacteria originating from the mouth). Unlike the situation with prosthetic heart valves, there is currently no evidence to suggest any link to IDPs and they should be considered low risk.^12^^,^^13^ In line with this, no guideline committees currently recommend AP for patients with stents unless other cardiac risk factors (eg a prosthetic heart valve) are also present to make them high risk.^12^^,^^13^

### Patients at low or unknown risk of IE

The risk of developing IE following an IDP (Box 1) is extremely low for the vast majority of the population (ie those at low/unknown risk) even without AP (see Table 1 in accompanying article).^9^Two recent US studies showed that the incidence of IE following an IDP without AP cover was just 3/million procedures in those with employer-provided medical/dental cover^14^ and 15/million procedures in those with Medicaid cover.^15^ Moreover, AP was of no benefit in preventing IE in those at low/unknown risk of IE.^14^^,^^15^^,^^16^

The slightly higher incidence of IE following IDPs in those with Medicaid cover is likely to reflect worse oral hygiene, limited access to, and more rudimentary, dental care, and higher rates of injection drug use.^15^ Nonetheless, given the low incidence of IE following IDPs in those at low/unknown IE risk, it would be reasonable to argue that the risk of IE is so low for the vast majority of the population as to not pose a ‘material risk'. In other words, the risk is too low under the new rules on consent for clinicians to be required to inform the patient.

### Identify all patients at increased IE risk

**Fig. 1:**
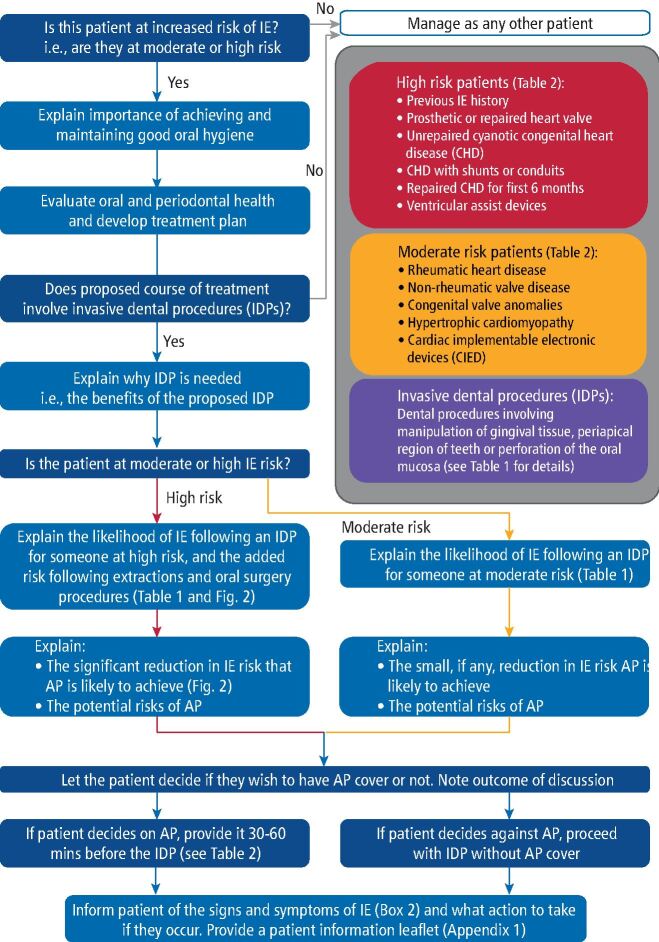
Algorithm for the management of infective endocarditis

The same is not true, however, for patients with predisposing cardiac conditions that place them at increased risk of IE and identification of these individuals is essential (see Table 1 in accompanying article).^9^ In the light of new evidence, we provide an outline for the management of patients at increased risk of IE (Fig. 1) until better guidance is provided by NICE. If there is doubt about the risk status of an individual patient, this should be clarified with their cardiologist or cardiac surgeon.

### Moderate risk

The two recent US studies showed that the risk of IE following IDPs without AP cover in moderate-risk patients (see Table 1 in accompanying article)^9^ was 23 IE cases/million IDPs amongst those with employer-provided medical/dental insurance (ie ~8 times higher than those at low/unknown risk),^14^ and 160 IE cases/million IDPs (ie ~11 times higher) in those with Medicaid cover.^15^ Those at moderate IE risk are, therefore, at 8-11 times greater risk of developing IE following IDPs than the majority of the population. Whilst still low, this could be considered of material importance, meaning that the risk (and its management) should be discussed prior to IDPs in this group of patients.

### High risk

The two US studies showed a much higher incidence of IE following IDPs in high-risk patients (see Table 1 in accompanying article)^9^ without AP cover (1,009 cases of IE/million IDPs in those with employer-provided medical/dental insurance and 5,156 IE case/million IDPs in those with Medicaid cover).^14^^,^^15^ This group, therefore, have a 1-in-200-1,000 overall chance of developing IE following IDPs and are ~340 times more likely to develop IE following an IDP than the majority of the population. High-risk patients are therefore at materially increased risk of developing IE following IDPs and this risk (and means for its reduction) should be explained before any IDPs.

The incidence of IE was even higher following extractions and oral surgical procedures in high-risk patients without AP cover (8,968/million extractions and 24,043/million oral surgical procedures in those with employer-provided medical/dental insurance, and 9,828/million extractions and 23,980/million oral surgical procedures in Medicaid patients).^14^^,^^15^ High-risk patients, therefore, have an almost 1-in-100 chance of developing IE following dental extractions and an almost 1-in-40 chance after oral surgery.

## Advice for patients at increased (high/moderate) IE risk

Recent data strongly suggest that poor oral hygiene increases the risk of IE in those at moderate and high risk^17^^,^^18^ and these patients should be aware of the importance of achieving and maintaining good oral hygiene through regular professional and personal dental care.^18^ Recommended measures include oral hygiene instruction, advice on adjunctive oral hygiene procedures and regular scaling and polishing. However, it should be remembered that scaling is an IDP, requiring discussion of the risks and benefits of the procedure and use of AP.

## Consent to perform invasive dental procedures in those at increased IE risk

**Fig. 2:**
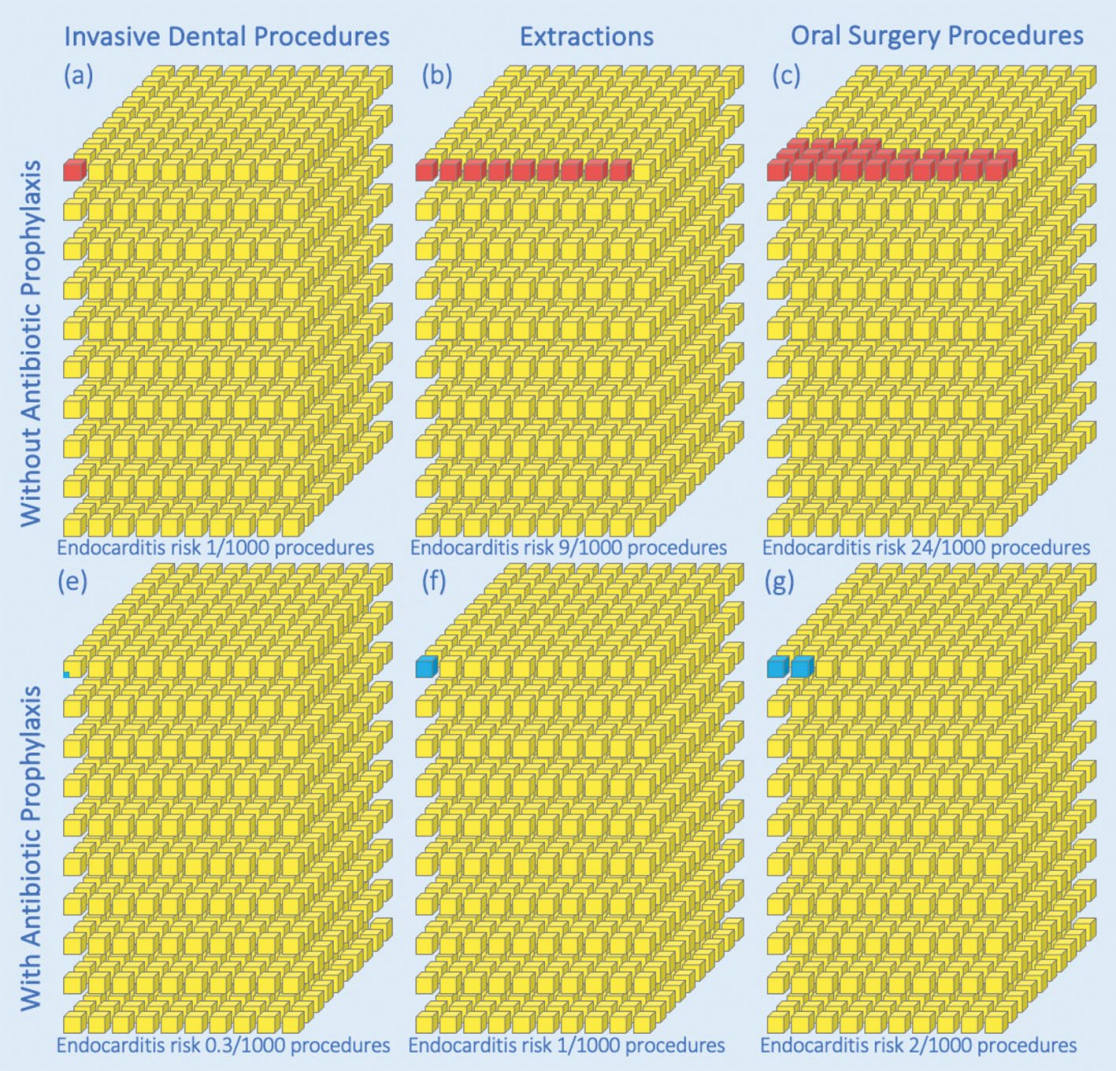
Diagram to facilitate explanation of the effect of AP in reducing the risk of IE following IDPs performed in high-risk patients. Each stack contains 1,000 yellow blocks representing 1,000 high-risk individuals undergoing IDPs (of all types), dental extractions or oral surgical procedures. Red blocks represent the number of individuals within each 1,000 population who would develop IE following the procedure in the absence of antibiotic cover (AP) (upper row). Blue blocks represent the number of individuals within each 1,000 population that would develop IE if each received AP before the procedure (lower row). Note: the incidence of IE following invasive dental procedures covered by AP is less than 1:1,000 (three in 10,000). The risk of a non-fatal adverse reaction following AP is even lower still (two in 100,000) and too small to feature in this figure. Data shown here are derived from a US study of patients with employer-provided medical/dental insurance cover.^14^ A similar study in Medicaid patients found generally higher values and it is therefore possible that the values shown are under-estimated for some patient groups.^15^ However, AP was equally effective in reducing the incidence of IE in both studies

All moderate- and high-risk patients should be advised of their risk of developing IE following an IDP and provide informed consent for any proposed procedure. Table 1 provides a visual information on the relative risk of developing IE after IDPs and may be useful to guide discussions with patients. Data for this (and Fig. 2) are from the US study of patients with employer-provided medical/dental insurance cover and may be an under-estimate (since the corresponding Medicaid data demonstrated a higher incidence of IE following IDPs).

**Table 1:** Information to facilitate explanation of the risk of different patient populations developing IE after invasive dental procedures. Data concerning the number of IE cases/million procedures are derived from a US study of patients with employer-provided medical/dental insurance cover.^14^ A similar study in Medicaid patients found generally higher values and it is therefore possible that the values shown are under-estimated for some patient groups^15^

Patient level of IE risk	Type of dental procedure	IE cases/million procedures^14^	Approximate risk of developing IE	Equivalent to
Low	Invasive dental procedures (as a whole)	3/million	1 in 333,000	1 person in 4 full Wembley stadiums
Moderate/intermediate	Invasive dental procedures (as a whole)	23/million	1 in 50,000	2 people in a full Wembley stadium
High	Invasive dental procedures (as a whole)	1,009/million	1 in 1,000	1 person in the largest commercial jet
High	Extractions	8,968/million	1 in 100	1 person in a large double-decker bus
High	Oral surgery procedures	24,043/million	1 in 40	1 person in a single-decker bus

## Advice concerning AP cover for IDPs

Patients should be told about the benefits and disadvantages of AP cover for IDPs.

### Benefits

Current data from large studies suggest that AP has no significant benefit in reducing the risk of IE following IDPs in those at low/unknown IE risk.^14^^,^^15^^,^^16^ Indeed, the risk of developing IE is lower (5-15 IE cases/million IDPs) than the risk of an adverse drug reaction (ADR) (23/million).

The risk of developing IE following IDPs is low for most moderate-risk patients (23-160 IE cases/million IDPs) and not much higher than the risk of an ADR (23/million). Recent studies have shown no (or negligible) benefit associated with use of AP to reduce the risk of IE following IDPs in this population.^14^^,^^15^^,^^16^ Therefore, AP is not routinely recommended by the AHA or ESC for moderate-risk patients. However, as mentioned in the recently updated ESC guidelines,^19^ there may be specific individual circumstances where AP could be considered for individual moderate-risk patients, including the presence of complex cardiac risk factors (eg presence of more than one moderate-risk cardiac condition) or comorbidities (eg diabetes mellitus, immune compromise, or renal dialysis). In these circumstances, the patient's physician, cardiologist or cardiothoracic surgeon may feel that a particular moderate-risk patient would benefit from AP and this information should be relayed to the patient and their dentist.

The risk of developing IE following an IDP is substantial in high-risk patients. In the US study of patients with employer-provided medical/dental cover, incidence of IE following IDPs was 1,009/million procedures (~1/1,000) without AP cover and even higher (5,156/million procedures; ~1/200) in Medicaid patients,^14^^,^^15^ far exceeding the risk of an ADR with AP (23/million).^20^ The IE risk following extractions and oral surgical procedures is even higher (Figure 2, Table 1). Fortunately, AP significantly reduces the incidence of IE following IDPs in this group (from 1/1,000 IDPs to ~1/2,800 [65% reduction] in those with employer-provided medical/dental insurance and from 1/200 to ~1/1,000 [79% reduction] in Medicaid patients). These changes are even more pronounced following extractions (1/100 to ~1/1,000 [90% reduction]) and oral surgical procedures (1/40 to ~1/500 [92% reduction]). The benefits of AP in reducing the incidence of post-procedural IE are therefore substantial in high-risk patients.^21^

Figure 2 illustrates the effect of AP in reducing the incidence of IE and may be helpful in discussions with patients.

The number needed to prevent (NNP) (the number of IDPs that would need AP cover to prevent one IE case) is another way of looking at the likely benefit of AP that may be helpful in discussions with high-risk patients. The lower the NNP, the more effective the prevention. For IDPs as a whole, the NNP for those with employer-provided medical/dental insurance and Medicaid cover was 1,536 and 244, respectively (extractions: 125 and 143, respectively; oral surgery: 45 and 71, respectively).

### Disadvantages

The principal disadvantage to the patient is the risk of an ADR; although, the risk associated with a single oral dose of amoxicillin is extremely low. UK adverse reaction data have recorded no fatal ADRs following a single 3 g oral dose of amoxicillin^20^^,^^22^ and the incidence of non-fatal ADRs of sufficient significance to merit reporting was only 23/million prescriptions.^20^ The risk of reportable fatal and non-fatal ADRs associated with clindamycin AP was much higher, however, and most guideline committees no longer recommend clindamycin AP for patients with a history of penicillin allergy for this reason. The ADR risk associated with currently recommended alternatives to amoxicillin (Table 2) is unknown. Other societal disadvantages to AP include the emergence of antibiotic-resistant organisms (although this risk is lower with single high-dose antibiotic use than with prolonged courses of antibiotics at sub-therapeutic doses or with suboptimal efficacy for the target organism), and cost (although AP has been shown to be highly cost-effective in high-risk patients).^21^

**Table 2:** AP regimens recommended for high-risk dental procedures in high-risk patients (based on 2021 AHA and 2023 ESC guidelines)^19^^,^^24^

Situation	Antibiotic	Single-dose 30-60 minutes before procedure	
		Adults	Children
No allergy to penicillin or ampicillin	Amoxicillin^1^	2 g orally	50 mg/kg orally
	Ampicillin	2 g IM or IV	50 mg/kg IM or IV
	Cefazolin or ceftriaxone	1 g IM or IV	50 mg/kg IM or IV
Allergy to penicillin or ampicillin^4^	Cephalexin^2^^,^^3^	2 g orally	50 mg/kg orally
	Azithromycin or clarithromycin	500 mg orally	15 mg/kg orally
	Doxycycline	100 mg orally	<45 kg, 2.2 mg/Kg
			>45 kg, 100 mg
	Cefazolin or ceftriaxone^3^	1 g IM or IV	50 mg/kg IM or IV
Key: IM = intramuscular. IV = intravenous. Notes: 1) In the UK, a practical alternative is the 3 g amoxicillin oral powder sachet that is made up with water and specifically available for this purpose. 2) Or other first- or second-generation oral cephalosporin in equivalent dose. 3) Cephalosporins should not be used in patients with a history of anaphylaxis, angioedema or urticaria after penicillin or ampicillin (due to the risk of cross-sensitivity). 4) Clindamycin is no longer recommended as AP for a dental procedure.			

The risk of developing an ADR to amoxicillin (just 23 ADR/million AP prescriptions) is too small to be shown in Fig. 2. Moreover, none of these ADRs would be fatal. In contrast, ~30% of IE cases are fatal within one year of diagnosis.

Patients should be allowed to decide if they wish to receive AP following discussion of these benefits and disadvantages, and the outcome of these discussions should be recorded in the clinical notes.

## Suggested antibiotic regimes

Even though NICE guidelines acknowledge that some patients may benefit from AP, they provide no information about which AP regimen to use, whilst Scottish Dental Clinical Effectiveness Programme recommendations are now out of date.^23^ Most guideline committees (including the AHA and ESC) no longer recommend the use of clindamycin as an alternative to amoxicillin for AP in those with a history of penicillin allergy,^19^^,^^24^ owing to the risk of ADR, particularly Clostridioides difficile infections, even with the single 600 mg oral dose previously recommended for AP.^20^ Clindamycin AP has an ADR rate of at least 13 fatal and 149 non-fatal ADR/million prescriptions.^20^

Guidelines now recommend 2 g of oral amoxicillin 30-60 minutes before the procedure as AP in those with no history of penicillin allergy (Table 2).^19^^,^^24^ In the UK, a 3 g sachet of sugar-free amoxicillin powder mixed in water and taken orally 30-60 minutess before the procedure has traditionally been used for AP purposes and is still available, and makes a good alternative. For those with a history of penicillin allergy, a single oral dose of cephalexin 2 g, azithromycin 500 mg, clarithromycin 500 mg, or doxycycline 100 mg 30-60 minutes before the procedure is recommended by the ESC and AHA (Table 2).^19^^,^^24^

A UK study of the use of a single oral 3 g sachet of amoxicillin demonstrated a non-significant increase in the number of resistant streptococci by day three that returned to baseline within 21 days. When given at weekly intervals, the numbers of resistant OVGS increased significantly after the second and third doses of amoxicillin and persisted for 4-7 weeks. To prevent the development of antibiotic resistance with repeated AP use, these authors suggested that for high-risk patients requiring repeated IDPs, either an alternative antibiotic should be used each time (see Table 2 for alternatives), or there should be intervals of at least four weeks between treatment sessions.^25^

## Advice to patients on recognising IE

The possibility of IE is not eliminated by AP and all moderate- and high-risk patients who have undergone IDPs (whether covered by AP or not) should be informed of the symptoms of IE (Box 2) and the action needed in the event that they arise. This is extremely important since early diagnosis substantially improves clinical outcomes for patients. Symptoms of IE caused by oral bacteria may arise soon after the procedure but can be delayed for weeks. Patients should be warned that they should seek medical assessment at the earliest opportunity if symptoms occur and ensure that their general practitioner is aware of any recent IDP and their increased risk of IE. This information may best be provided in the form of a patient information leaflet (Appendix 1). Ideally, the patient's cardiologist, cardiothoracic surgeon, or physician will already have provided the patient with this information (so that the dentist only has to provide a reminder), but this is not always the case.

Box 2 Symptoms of infective endocarditis

- High temperature (38 ℃ or above)
- Night sweats
- Shortness of breath on exertion
- Tiredness (fatigue)
- Muscle and joint pains
- Unexplained weight loss
- Loss of appetite.

**Other presentations:**

- New or changing heart murmur
- Spotty red skin rash (petechiae)
- Narrow, reddish-brown streaks under the nails (splinter haemorrhages)
- Red tender lesions on the fingers or toes (Osler's nodes)
- Confusion
- Stroke.

## Conclusions and call for action

The current situation is complex and unsatisfactory for clinicians and patients alike. New evidence (reviewed in the accompanying article in this issue)^9^ provides data to inform patients and clinicians about the risk of IE posed to different groups of patients by IDPs and steps to mitigate this risk. Based on this evidence, the 2023 ESC guidelines provide clear recommendations on the management of such patients. As we await new and updated NICE guidance, we hope that this article provides useful information for dental practitioners, many of whom remain puzzled and confused by (or even unaware of) the UK's isolated stance.

**Figure:**
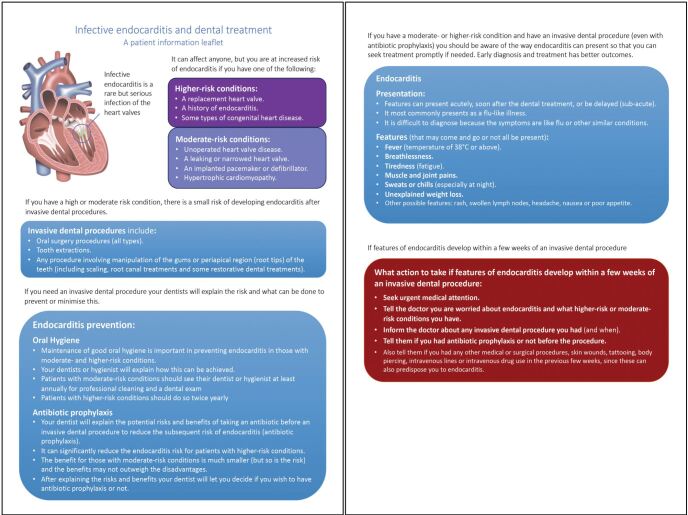
Appendix 1 Patient information leaflet. Heart image courtesy of iStock, credit: sajithsaam
